# The complete chloroplast genome of a medical herb, *Gentianopsis paludosa* (Hook. f.) Ma (Gentianaceae), from Qinghai-Tibet Plateau in China

**DOI:** 10.1080/23802359.2021.1915198

**Published:** 2022-08-12

**Authors:** Hongyu Wang, Jinping Li, Yang Zeng, Ruifeng Zhang, Junyan Zhang, Likuan Liu

**Affiliations:** a The College of Biological Science, Qinghai Normal University, Xining, China; b Academy of Plateau Science and Sustainability, Xining, China

**Keywords:** *Gentianopsis paludosa*, chloroplast genome, Gentianaceae, Qinghai-Tibet Plateau, phylogenetic trees

## Abstract

*Gentianopsis paludosa* (Hook. f.) Ma (Gentianaceae) is one of the genuine medicinal materials in Qinghai-Tibet Plateau, China. Here we report the first chloroplast (cp) genome of *G. paludosa* using Illumina NovaSeq 6000 platform. The length of its complete cp genome is 151,568 bp, containing four sub-regions; a large single copy region (LSC) of 82,834 bp and a small single copy region (SSC) of 17,928 bp are separated by a pair of inverted repeat regions (IRs) of 25,403bp. The complete cp genome of *G. paludosa* contains 130 genes, including 85 protein-coding genes, 37 tRNA genes, and 8 rRNA genes. The overall GC content of the cp genome is 37.8%. The phylogenetic analysis, based on 23 cp genomes, suggested that *G. paludosa* is closely related to *G. grandis* (H. Smith) Ma and *Swertia* species.

Gentianopsis Ma (Gentianaceae) includes about 24 species distributed in the Asia, Europe, North America. There are only five Gentianopsis *species* distributed in China (He 1988). *G. paludosa*, known as the ‘Tibetan capillaris’, using whole herb as medicine, is one of the common Tibetan herbal medicines used in the treatment of gallbladder and liver diseases (Song 1986). It is mainly distributed in flood land, forest margins and meadows area (altitude 1180–4600 m) in Tibet, Qinghai, Sichuan, Yunnan Provinces and other places in north of China (Yang 1991; He 1988).

To study the systematic position and genetic background of *G. paludosa*, we sequenced the *G. paludosa* DNA and obtained its complete chloroplast (cp) genome. The voucher specimen of *G. paludosa* was collected from the riverside in the Yuer village in Xianmi township, Menyuan County, Qinghai Province, China, on August 11 in 2019 (alt. 2683 m, E101°58′6.69″, N37°16′41.76″), and the specimen deposited at Herbarium, School of Life Sciences, Zhengzhou University, the voucher number is ZZU2019-6306. The total DNA was isolated from leaf materials of the voucher specimen using the plant genomic DNA extraction kit (Solarbio LIFE SCIENCES, China). The DNA concentration and quality were then measured by NanoDrop2000c micro-uv spectrophotometer (Thermo Scientific, America). The DNA was sequenced at Novogene Biotech Co. (Beijing, China) using the Illumina NovaSeq 6000 platform with a 150-bp shotgun library. Finally, 2.34 G of 150-bp paired-end raw reads of *G. paludosa* were obtained, processed and assembled following the method of Nicolas et al. (2017). The raw sequencing reads is deposited in SRA, and the number is PRJNA656080. The assembled contigs were mapped to the reference cp genome (*G. grandis*, GenBank accession no. NC_049879) and annotated using Geneious Prime software(https://www.geneious.com). The border regions between the large single copy region (LSC), the small single copy region (SSC) and two inverted repeat regions (IRs) were validated by PCR amplifications and Sanger sequencing. The complete cp genome of *G. paludosa* is 151,568bp in length, and it released to NCBI (GenBank accession no.MT921831). It contains two IRs of 25,403 bp, separated by a LSC of 82,834bp and a small SSC of 17,928bp. The cp genome of *G. paludosa* is comprised of 130 genes, including 85 protein-coding genes, 8 rRNA genes, and 37 tRNA genes. The overall GC content of the cp genome is 37.8%, while the corresponding values of the LSC, SSC, and IR regions are 35.8%, 31.8%, and 43.3%.

The cp genome of *G. paludosa* and 22 cp genome sequences (downloaded from GenBank) were aligned using MAFFT (Katoh and Standley 2013) and then constructed phylogenetic trees using neighbor-joining (NJ) and maximum likelihood (ML) methods in MEGA7 (Kumar et al. 2016). *Rhodiola rosea* (Crassulaceae) and *Haloxylon persicum* (Chenopodiaceae) were selected as outgroups. The results showed that *G. paludosa* was sister group to *G. grandis* and they were close related to *Halenia corniculata* (Figure 1). The phylogenetic analysis was consistent with previous studies (Park et al. 2019; Chassot et al. 2001).

**Figure 1.**
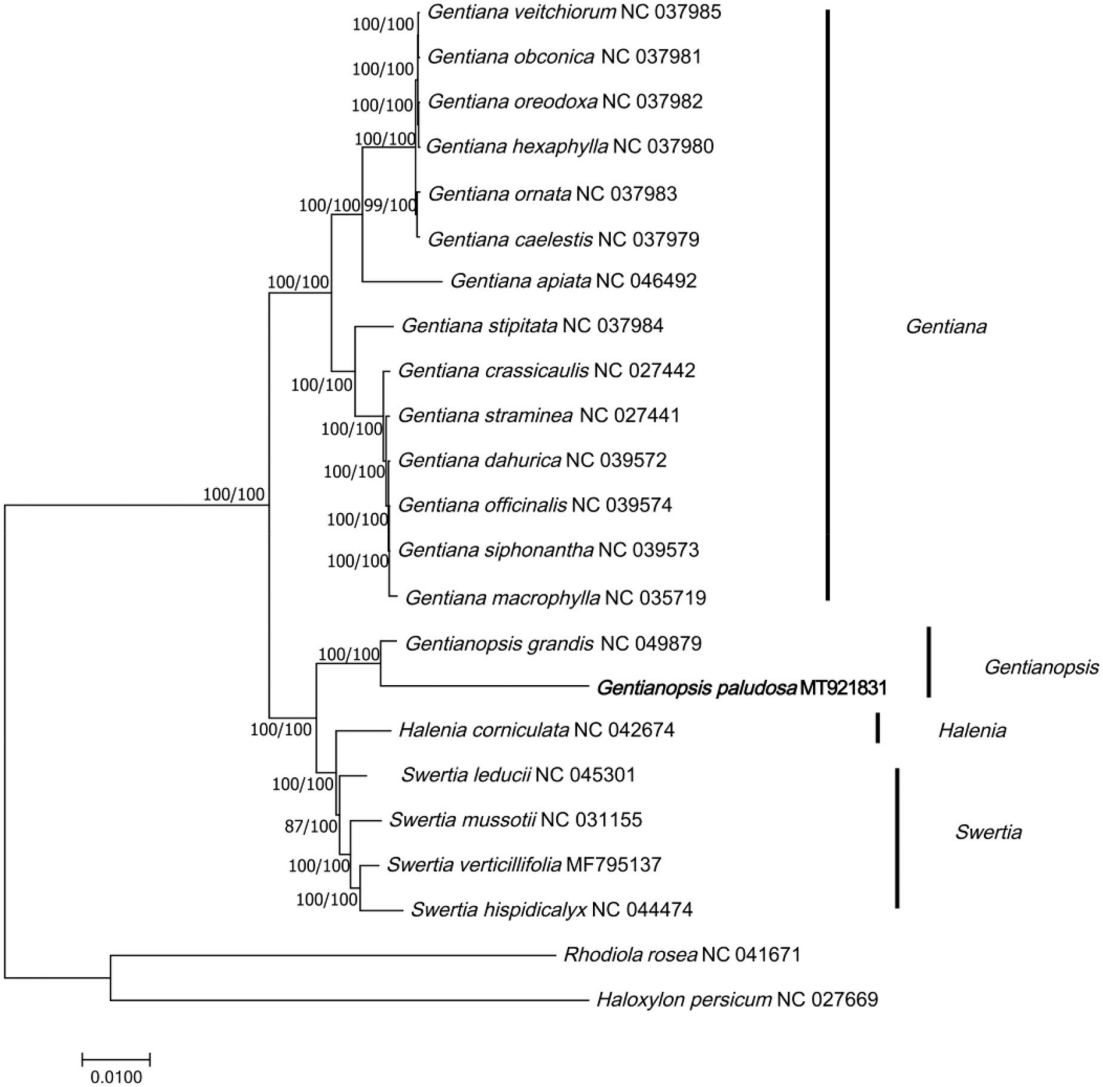
Phylogenetic tree of 23 species based on complete chloroplast genome sequences using NJ (with 1000 replicates) and ML (with 1000 replicates) methods. The numbers below the branches indicate the corresponding bootstrap support values from the ML and NJ trees. *Haloxylon persicum* (NC_027669) and *Rhodiola rosea* (NC_041671) are outgroups.

## Data Availability

The data that support the findings of this study are openly available in GenBank of NCBI at (https://www.ncbi.nlm.nih.gov/), under the accession no. MT921831. The associated BioProject, SRA, and Bio-Sample numbers are PRJNA656080, SRR13089735, and SAMN15770945 respectively.
